# Garniss Curtis (1919–2012): Dating Our Past

**DOI:** 10.1371/journal.pbio.1001650

**Published:** 2013-09-10

**Authors:** William Henry Gilbert

**Affiliations:** Associate Professor, Department of Anthropology, California State University, East Bay, Hayward, California, United States of America; Researcher, Human Evolutionary Research Center, University of California, Berkeley, Berkeley, California, United States of America

## Abstract

Henry Gilbert pays tribute to a pioneer who enlisted radioactive decay to target evolutionary questions.



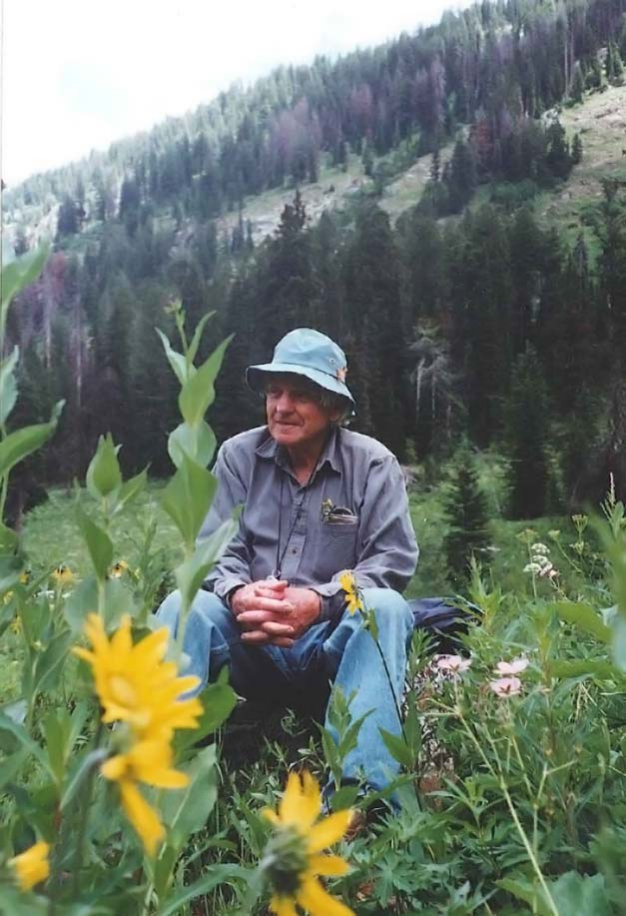
Garniss Curtis in the Wyoming backcountry, 2001.


Around the time that *On the Origin of Species* was published, Lord Kelvin authoritatively stated that the Earth was between 20 and 400 million years old, a range still quoted today by many who deny evolution. As it was difficult to conceive of life's diversity arising via natural selection and speciation in so short a span, the apparent young Earth formed a serious barrier to the plausibility of evolution's capacity to generate the tree of life. T. H. Huxley famously attacked Kelvin, saying that his calculations *appeared* accurate due to their internal precision, but were based on faulty underlying assumptions about the nature of physics [1].

How right Huxley was.

Garniss Curtis was born in San Rafael, California in 1919. This was just 15 years after Ernest Rutherford, famous for discovering the nucleus of the atom and the existence of the phenomenon of radioactive half-life, walked into a dimly lit room to announce a new date for the age of the earth: 1.3 billion years. Lord Kelvin, the venerable alpha of Earth-age estimates, was in attendance.

*“I came into the room, which was half dark, and presently spotted Lord Kelvin in the audience and realized that I was in trouble at the last part of my speech dealing with the age of the Earth, where my views conflicted with his. To my relief, Kelvin fell fast asleep, but as I came to the important point, I saw the old bird sit up, open an eye, and cock a baleful glance at me! Then a sudden inspiration came, and I said, ‘Lord Kelvin had limited the age of the earth, provided no new source of heat was discovered. That prophetic utterance refers to what we are now considering tonight, radium!’ Behold! the old boy beamed upon me.”* —Ernest Rutherford [2]

Although not Rutherford's primary aim, his work contributed to our understanding of biological evolution by ushering in a sensible, realistic temporal framework for Earth's billions of years that was more obviously compatible with Darwinian evolution than Kelvin's young estimate was. Garniss, who passed away on December 18, 2012 at age 93, would follow Rutherford in applying knowledge of radioactive decay to help settle questions about key dates in Earth's history, but he would more actively target evolutionary questions. Unfortunately, Rutherford's work with radium decay did little to provide actual ages for fossils due to the rarity of rocks dateable with the method and several factors that made it extremely imprecise. Garniss and colleagues from the University of California, Berkeley transformed the field by recognizing that the steady decay of radioactive potassium to argon in volcanic lava or ash after an eruption could be measured using a mass spectrometer to provide a date for the eruption with a tiny fraction of the error inherent to Rutherford's methods. Just as importantly, potassium-argon dating could be applied to minerals very common in fossil-bearing units. And it worked on younger rocks, meaning it could be used to date the human fossil record.

Garniss' work had huge implications. His application was at the nexus of a profound change in humanity's self-perception and notions of equality. Garniss was part of an intellectual movement involving deep time and biological evolution that had gained an unstoppable inertia by the mid–20th century, becoming much larger than any one individual or discipline. Biological evolution had achieved overwhelming consensus in the scientific community while at the same time ascending to extreme societal controversy; broadly accepted fossil hominids had been discovered in Africa and Asia, and Earth's age was understood to be in the billions of years. But even with all this, many in the mid–20th century held pre-Darwinian notions of human uniqueness and racial differentiation. In the first two decades after World War II “race” was still a concept frequently employed by scientists, and many thought that “races” had existed for a very long time as unique entities (Figure 1). Additionally, before potassium-argon dating there were no reliable dates anywhere near the human-chimpanzee split. The “ape-men” from South Africa, while finally acknowledged as relevant to human evolution by mainstream scientists, were surrounded by a legacy of dating problems and stratigraphic confusion.

**Figure 1:**
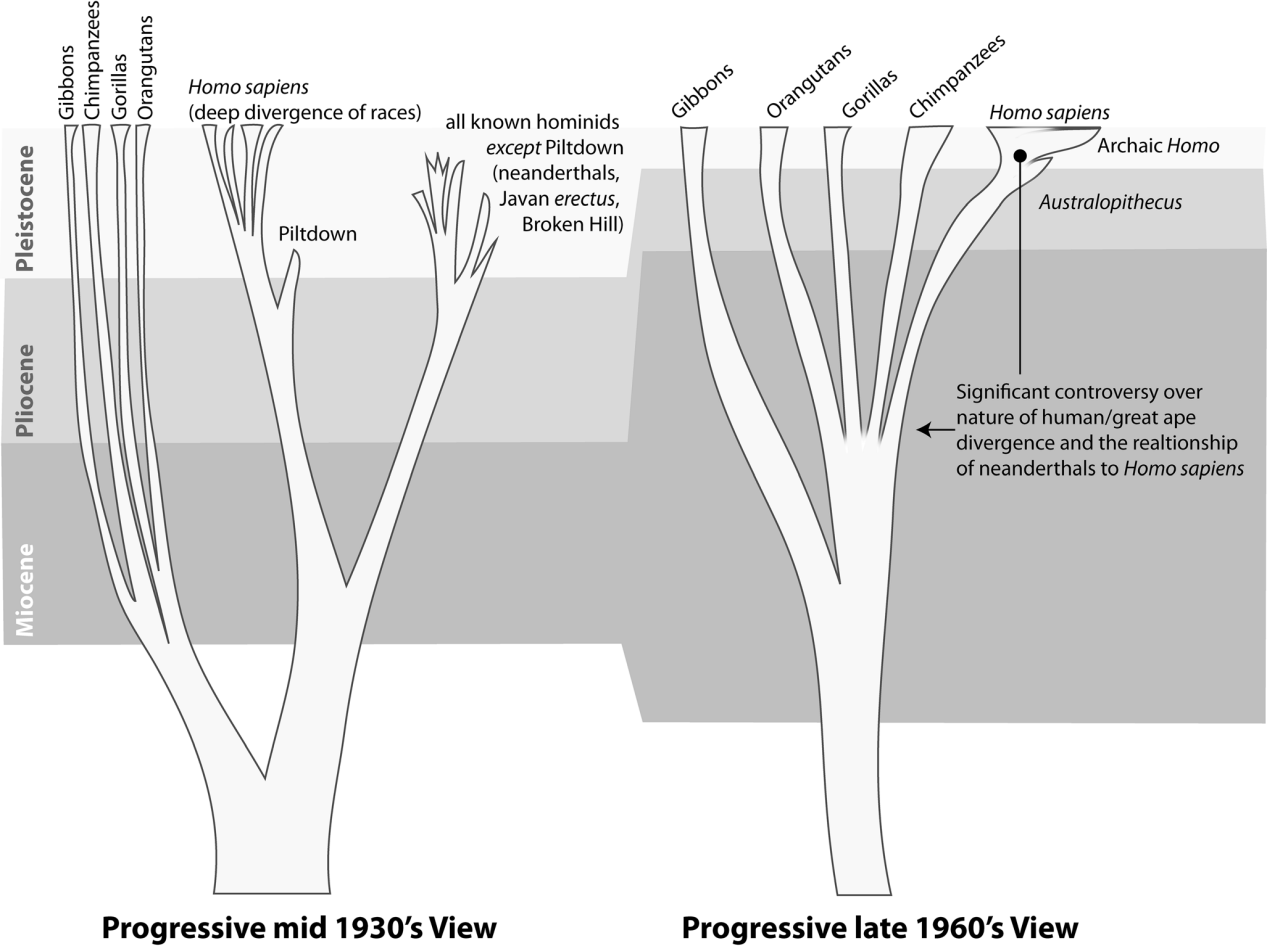
Early- [7] to mid- [8] 20th-century views of human evolution.

Garniss entered the stage 1959 as he undertook the dating of Bed I at Olduvai Gorge in Tanzania where robust *Australopithecus* skull OH 5 (originally called *Zinjanthropus boisei* or “Zinj”), the first major human ancestor cranium found in the African Rift Valley, had recently been discovered by Louis Leakey's project. The Leakeys are now famous for popularizing human origins research in the late–20th century, but it is with Garniss' dates that this notoriety was insured. The best guess for the age of OH 5 had been around 500,000 years old [3]. Garniss and Jack Evernden showed that its age was actually closer to 2 million. “One thing is certain,” Garniss wrote at the time. “Olduvai Man is old, old, old!” [4]

Garniss was field-oriented and critical. He did not promote lab-perfect dates if they were misapplied. “OH 9 could just as easily have come from Bed IV as Bed II. It was in the bottom of a gully,” Garniss once told me in reference to a *Homo erectus* calvaria (partial cranium) from Olduvai Gorge. The distinction was important because most experts assume the calvaria was from Bed II, hundreds of thousands of years older than Bed IV. Garniss suspected the calvaria could be much younger *because he was there*. And “being there” is what made this amazing man the conduit of knowledge that he was. Although it may seem a laboratory-specific endeavor, dating volcanic tuffs and the fossils and artifacts they bound takes much more than a mass spectrometer, a vacuum furnace, and a scientist in a lab coat. The lab work is certainly an essential part of things—and Garniss did it well—but there are countless non-intuitive depositional environments and processes associated with volcanism: violently projected air-fall ashes, massive floods of lava, ferocious flows of superheated, liquefied ash surging across large landscapes, pumices floating down ash-choked mud streams, and all sorts of waterways carrying and depositing ash-laden silts. Volcanic deposits are famously easy to misinterpret in the field. Replicability in radiometric dating thus means much more than just sending multiple samples of the datable ash through competing labs; it means that the sample must first be properly collected and interpreted in its place, a crucial aspect of the job invisible to most who don't do fieldwork. One of many examples of Garniss' dedication to fieldwork and its role in geological interpretations was his work in the Valley of Ten Thousand Smokes (Alaska) starting in 1953, only a geological instant after the Mount Katmai eruption of June 6, 1912. He observed, walked on, touched, sampled, analyzed, and probably even tasted the consequences of a major, photo-documented 20th-century volcanic eruption. He knew what a big volcanic event did to a depositional landscape and the manifold stratigraphic consequences. Such events are complex, and Garniss was not the type amenable to armchair inductive reasoning based on a few observations or samples.

The effect that potassium-argon dating had on human origins research was profound. Before Garniss applied potassium-argon dating, reliable dates for hominid fossils were rare. None were precise and none were in Africa, the continent of our early ancestors. Nobody knew how old *Australopithecus* was, and nobody suspected the very recent origin of *Homo sapiens*. As it happened, Garniss brought together the skill, intelligence, and determination that helped resolve these fundamental questions. In the early 1960s he worked with colleagues Jack Evernden, Don Savage, and Gideon James to give absolute numbers to the previously undated North American land mammal record using the potassium-argon method [5]. This paved the way for seeing an evolutionary pattern in mammals on a vastly different scale than had previously been available, dramatically enhancing the information that could be extracted from the fossil record. Rates of evolutionary adaptation, the nature of speciations and adaptive radiations, global paleobiogeography (migrations, etc.), and many other evolutionary patterns and processes moved from conjecture to testability.

Garniss was not afraid to ruffle a few feathers. Even the scientific celebrities whose feet of clay Garniss revealed ultimately feared and respected his allegiance to the scientific method. This is exemplified in the way he parted collaboration with the Leakeys, one generation at a time. After working on dating *Australopithecus* and early *Homo* at Olduvai, Garniss argued with Louis Leakey over dates for Rusinga Island “*Kenyapithecus” africanus*. Leakey said they were over 30 million years old, but Garniss dated volcanic rocks from the locality and said they were about 17 million years old, accusing Leakey of letting his preconceived notion of a very early genus *Homo* shape his chronology [6]. Leakey promptly replaced Garniss with Jack Miller, but Miller had no way to credibly detect ages different from what Garniss had already found. Garniss is also famous for analyzing samples of the notorious KBS Tuff, an ash layer from Koobi Fora, Kenya at a site run by Louis Leakey's son Richard. Richard Leakey was promoting old ages (2.6 Ma) for the tuff and the *Homo* fossils they dated, but paleontologists were suspicious of biochronological evidence and suspected that the dates claimed by Leakey for the tuff were wrong [6]. The age estimate of approximately 1.8 Ma obtained by Garniss and colleagues indicated that the KBS Tuff was much younger than had been promoted, another serious blow to Leakey notions of an older genus *Homo*.

Garniss played a role in human origins research well into the 21st century, working directly on, among other things, dates for *Homo erectus* fossils in Java. During his long and illustrious career, he made many major contributions outside of human origins and evolutionary biology as well. Garniss continued to return to the Valley of Ten Thousand Smokes and Mount Katmai throughout his life. He was involved in understanding the formation of the Sutter Buttes (a small mountain range in Northern California), mapped parts of the Sierra Nevada mountain range, and was an expert on the formation of the local Berkeley Hills.

Garniss' UC Berkeley geochronology lab became the Berkeley Geochronology Center, which continues to serve as one of the world's foremost geochronology laboratories. The Center has dated a large percentage of the world's human fossil record, more than can be listed here, including famous hominids like Ardi and Lucy. Garniss mentored and was loved by many students, colleagues, and friends. One of his star students, Paul Renne, a UC Berkeley professor who now runs the Berkeley Geochronology Center, and several of Garniss' former colleagues have continued to develop argon-based dating procedures and apply them to a wide range of deep history questions.

Beyond his considerable scientific achievements, Garniss was a fine friend. He loved music, art, wine, and conversation. He was pleasant, respectful, intelligent, thoughtful, and critical. He was full of stories about people, the world, culture, scientific intrigue, and society. He was concerned with world events, and conversational on topics of philosophy and reality. He loved to put slightly outlandish ideas on the block for criticism. Any time spent with Garniss was rewarded with warm memories and plenty of enlightenment. Garniss was a key member of an internationally and topically diverse network of scholars and intellectuals, who are still adjusting to his departure.
